# Effect of Low Concentration Sn Doping on Optical Properties of CdS Films Grown by CBD Technique

**DOI:** 10.3390/ijms12096320

**Published:** 2011-09-23

**Authors:** Atefeh Jafari, Azmi Zakaria, Zahid Rizwan, Mohd Sabri Mohd Ghazali

**Affiliations:** 1 Advanced Materials and Nanotechnology Laboratory, Institute of Advanced Technology, Universiti Putra Malaysia, 43400 UPM Serdang, Selangor, Malaysia; E-Mail: atefeh.j87@gmail.com; 2 Department of Physics, Faculty of Science, Universiti Putra Malaysia, 43400 UPM Serdang, Selangor, Malaysia; E-Mail: mgm.sabri@gmail.com; 3 National Textile University, Sheikhupura Road, Faisalabad 37610, Pakistan; E-Mail: zahidrizwan64@gmail.com

**Keywords:** chemical bath deposition, doped CdS, solar cells, optical properties

## Abstract

Thin and transparent films of doped cadmium sulfide (CdS) were obtained on commercial glass substrates by Chemical Bath Deposition (CBD) technique. The films were doped with low concentration of Sn, and annealed in air at 300 °C for 45 min. The morphological characterization of the films with different amounts of dopant was made using SEM and EDAX analysis. Optical properties of the films were evaluated by measuring transmittance using the UV-vis spectrophotometer. A comparison of the results revealed that lower concentration of Sn doping improves transmittance of CdS films and makes them suitable for application as window layer of CdTe/CIGS solar cells.

## 1. Introduction

Cadmium sulfide (CdS) thin film has attracted increased attention in recent years because of its wide direct band gap energy, optical and electrical properties, and stability, which is suitable for application as a window layer in solar cells [1–3]. Various methods, such as chemical vapour deposition, sputtering and spray pyrolysis [4], chemical bath deposition (CBD) [5,6], close space sublimation (CSS), and successive ionic layer adsorption and reaction (SILAR) [7] have been used for depositing CdS films. CBD, also called Growth solution, is known as a low cost and facile method to raise the performance of CdS window used in Cadmium telluride (CdTe) and Copper indium gallium selenide (CIGS )solar cells. Deposition of CdS thin films by this technique is based on controlled precipitation of the material, wherein the release of Cd^2+^ ions is controlled by adding a complex agent to Cd salt. The S^2−^ ions are provided by a controlled hydrolysis and disintegration of thiourea. The reaction of both ions, aided by ammonia, produces a homogeneous layer of CdS onto the substrate. Ammonia will prevent the formation of undesirable solid phase and settling down by forming complexes with Cd ions [8–10].

Recently, the efficiency of CdS semiconductor film was improved by changing its optical and/or electrical properties by doping with some foreign elements such as Copper [11], Gallium [12], and Erbium [13], *etc*. Earlier, Sn doped CdS films, produced by using tartaric acid as the complexing agent, were less transparent [14]. Since the transmittance of light is the most important requirement of the window layer in a thin film structured solar cell, the search for methods to form very high transparent CdS films by doping Sn is highly warranted. In this paper, we present the fabrication of more transparent Sn doped CdS films at low dopant concentration by using CBD technique. Additionally, the optical, structural and morphological properties of the films are also presented.

## 2. Results and Discussion

Visibly, all the films prepared by CBD were mirror-like and showed good adhesion to the substrate. The color of films changed partially from yellow to dark yellow by increasing the copper content in solution.

**Figure 1:**
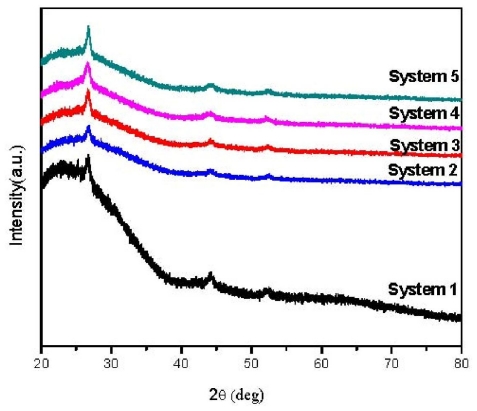
X-ray diffractograms of all systems.

Study of the morphology of films deposited by CBD showed they are polycrystalline nature (Figure 1). System 1 (undoped) shows peaks at 2θ = 26.5887, 30.9108, 44.1861, 52.2420 and 54.4284 (ref: 01-080-0019), which belong to (111), (200), (220), (311) and (222) of cubic CdS phase, respectively. These peaks also appeared in all doped film which indicates that Sn doping did not change the cubic CdS lattice structure. Films with lower Sn doping exhibited strong preferential orientation of (111) plane. The corresponding peak intensities from (111) plane increased with increasing Sn doping, which indicates that Sn atoms of slightly bigger radius are inserted at the smaller S atoms in the cubic lattice. Hence, the Sn ions substitute S in lattice positions in good order instead of filling the free spaces. Therefore, very low concentration of Sn dopants did not change the crystal structure of CdS; however, other peaks related to Sn compounds, mostly the SnS with orthorhombic structure, appeared in all doped films. Increasing Sn doping of CdS was accompanied by decrease in *d* spacing (see Table 2), indicating smaller cubic structure. The *d* values are in agreement with JCPDS standard. In the presence of low Sn doping also, the films produced a few additional small peaks at 2θ = 23.2035, 60.867 and 52.716 (ref: 01-079-2193) that belong to (111), (314) and (132) related to SnS with orthorhombic phase. These peaks did not show significant change in agreement with previous works [12,14].

The crystallite sizes, as determined by using Scherrer formula on the XRD patterns [15], increased from 18 nm for undoped CdS to 37 nm for CdS doped with Sn of 0.042 M concentration (Table 2). The small average crystallite size indicates that dominated deposition takes place by ion-by-ion condensation because of proper conditions of deposition and slow growth rate during the whole experiment. As a result, a much smaller, denser grain size and thinner films were achieved [16].

**Figure 2:**
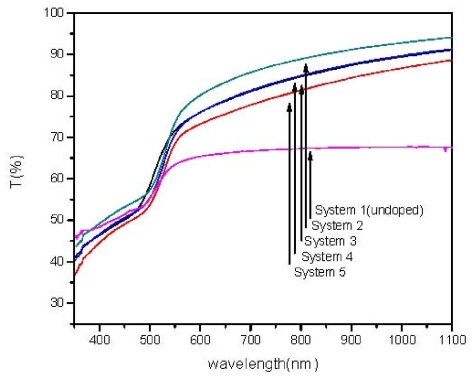
The transmittance spectra for all systems.

The UV-Vis transmission spectra of all the Sn-doped films exhibited sharp fall at the band edge, which suggests good crystallinity of the thin films (Figure 2). Films doped with Sn showed a sudden increase in transmittance because they are thicker and there is more space inside the lattices in doped films. However, in System 3 onwards, the transmittance spectra showed less transparency with increasing dopant, which agrees well with the earlier report on Copper doping [11]. This may be attributed to more scattering of photons by the introduction of dopant as foreign atoms, which may reduce transmittance. Compared with the constant value of 65% transmittance of undoped film before band edge, the doped films have about 90% transmittance of Incident Solar Spectrum around 1100 nm wavelength region and fall to a minimum of 73% before band edge. This is about a 20% improvement in transparency for the low Sn doped film.

**Figure 3:**
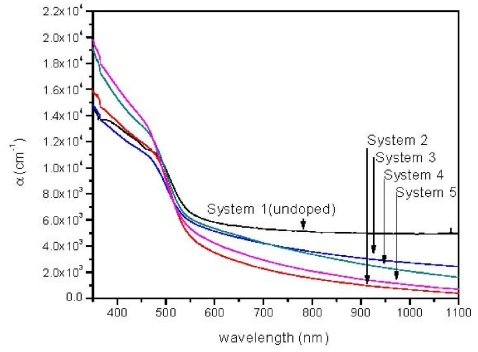
Absorption spectra for all systems.

The absorption spectra obtained from the transmission spectra of each sample is shown in Figure 3. Here, *α* value increases with the increase in Sn ions in growth chemical solution. All doped film spectra have lower *α* values compared to the undoped films.

**Figure 4:**
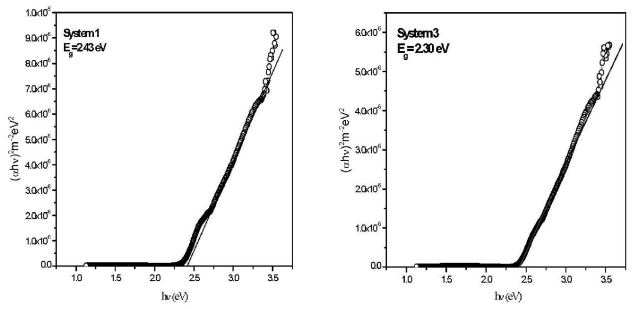
A typical plot of (*αhυ*)^2^ *versus* photon energy for Systems 1 (left panel) and 3 (right).

**Figure 5:**
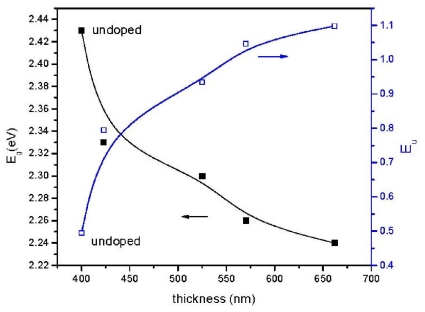
Variation of *E_g_* and *E_u_* as a function of film thickness.

A typical plot of (*αhυ*)^2^ *versus* photon energy, obtained from Figure 3 is shown in Figure 4 for Systems 1 and 3. For the Systems 1, 2, 3, 4, 5, the corresponding thicknesses are 400, 423, 525, 570, 662 nm, and the corresponding *E_g_*’s are 2.43, 2.33, 2.30, 2.26, 2.24 eV, respectively. The dependency of *E_g_* decreases with film thickness (Figure 5). These phenomena are attributed to the quantum size effect of the grain size that changes the thicknesses of films [17]. No absorption occurs below the band gap (Figure 3). Hence, the increment of extinction coefficient is due to scattering by dopant atoms on the film surface. Urbach energy (*E_u_*), known as band tail width, is due to the disorder in the thin film material. As shown (Figure 5), the variation of Urbach energy is opposite to *E_g_*. In other words, the *E_g_* decreases when there is an increase of band tail and vice versa.

**Figure 6:**
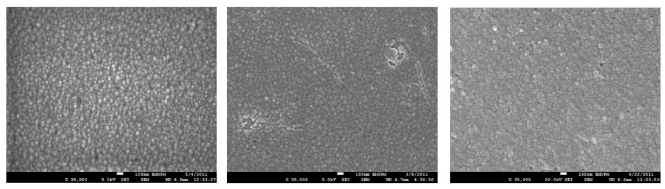
FE-SEM images of CdS films corresponding to System 1 (undoped; left panel), System 2 (middle panel), and System 5 (right).

**Figure 7:**
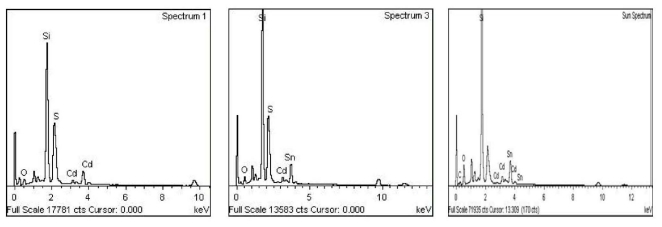
The EDAX spectra of CdS films for System 1 (undoped; left panel), System 2 (middle panel), and System 5(right panel).

Figure 6 presents FE-SEM micrographs of the film surfaces of System 1, 2 and 5. These images reveal that the films are compact, free of colloidal particles and are in good order. The sizes of grains were in the same range as those that show homogenous distribution of Sn dopant in films. Increasing the dopant led to grain growth. Both the doped films are more compact compared with the undoped film. During the growth of films, the nuclei appeared and grew as discrete surface grain. The surface grain size became larger, with less void area, and led to the formation of high quality film surfaces. The EDAX analysis of the undoped and two doped systems is shown in Figure 7. For undoped film, the maximum amount of Cd and S observed were 31.04% and 10.08%, respectively. For System 2 and 5, the quantities of Cd decreased, S remained almost constant, and Sn increased from (10.09:3.68:6.20) to (9.06:3.0:9.06). In both the systems, at low dopant concentration, the Sn atoms substitute Cd atoms in the lattice. Other systems, such as 3 and 4, showed variations intermediate to Systems 2 and 5.

## 3. Experiment

The films were grown on glass substrates in bath solution at 75 °C. Two separate solutions, one containing cadmium sulfate (CdSO_4_, 99.9%, Alfa Aesar) and the other containing thiourea (SC(NH_2_)_2_, 99%, Alfa Aesar) in deionized water were prepared first as sources of cadmium and sulfur, respectively. The concentration ratio of Cd:S of these two solutions was maintained at 1:2 for all depositions. Tin chloride (SnCl_2_) solution was used as a Sn dopant source. The films were grown on commercial glass slides, previously cleaned by ultrasonication in ethanol followed by acetone for 45 min, washed with deionized water and dried in an oven. For CBD, the solutions were first equilibrated in a water bath to reach a constant temperature of 75 °C. Ammonia as complexing agent was then added drop-by-drop into the CdSO_4_ solution to make it an alkaline solution with pH between 11 and 11.5. The concentrations of CdSO_4_, thiourea, and SnCl_2_ were kept constant at 0.005, 0.01, 0.0003 M, respectively.

Various volumes of Sn dopant solution (SnCl_2_) were added to four out of five equal portions of the CdSO_4_ solution, then the thiourea solution was added while continuously stirring. Details of the volumes of CdSO_4_, thiourea, SnCl_2_ or molar ratios of Cd, S, Sn used for CBD are presented in Table 1. Cleaned glass substrates were immersed vertically in the solution using special holders under stirring condition. Many deposition parameters such as temperature [18], deposition time [19], stirring speed and pH, that may affect the films, were kept constant during the growth of the films. The molar ratios of Sn dopant solution were varied carefully to achieve a precise amount of doping. The beaker was covered to avoid ammonia evaporation that can result in pH reduction. The samples were taken out after 40 min, rinsed ultrasonically in distilled water, and annealed at 300 °C in air for 45 min.

Optical properties of the films were measured by a UV-vis spectrophotometer (SHIMADZU UV-1650PC) in the wavelength range of 350–1100 nm. A PANalytical (Philips) X’Pert Pro PW1830 was used for XRD analysis (λ = 1.540598 Å) and the data were analyzed by X’Pert High Score software for identification of the crystalline phases in the films. The film thickness was measured by a high surface profile meter (XP-200, AMBIOS Technology). The surface morphology of the films were characterized using a Field Emission Scanning Electron Microscope (FE-SEM, JEOL-JSM 7200). The elemental compositions of the films were determined by the Energy Dispersive X-ray (EDAX) analysis.

The average interplanner distance *d* can be calculated from:

(1) $$\frac{1}{d^{2}}=\frac{h^{2}+k^{2}+l^{2}}{a^{2}}(\text{Cubic phase})$$

where, *h*, *k* and *l* are the Miller indices, *a* is the lattice parameter. The extinction coefficient, *α*, which include the addition of absorption and scattering coefficients is given by:

(2) $$\alpha =\frac{-\text{ln}(T)}{d}$$

where, *T* and *d* are the transmittance and thickness of the film, respectively. The optical band gap energy, *E_g_* of a material has a relationship as follows [20]:

(3) $$(\alpha h\upsilon )=A{\left(hv-E_{g}\right)}^{n}$$

where, *hυ* is the photon energy, *A* is a constant and *n* is considered as 1/2 for a direct transition. *E_g_* can be obtained by plotting a function of (*αhυ*)^2^ *vs.* photon energy, *hυ*, and extrapolating the linear part of the curve cutting the photon energy axis at (*αhυ*)^2^ = 0. The extinction coefficient displays a tail, which can be calculated by:

(4) $$\alpha =\alpha_{0}e^{\frac{hv}{E_{u}}}$$

where, *α_0_* is the constant. The inverse of the slope from the plot of ln(*α*) against photon energy gives the value of Urbach energy (*E_u_*) [21], which indicate disorder.

## 4. Conclusions

CdS thin films, doped with different amounts of Sn dopant have been prepared successfully by CBD technique. All films are polycrystalline in nature and consist of grains with cubic phase of CdS. During doping, Sn ions were found to substitute the Cd atom in the lattice without changing the crystal structure of the films. The transparency CdS film was found to increase from 20% without doping to a value of 90% after low concentration Sn doping in molar ratios 0.003:1 with respect to S. The transparency accompanied with band gap drops with further increase in doping concentration. The high transparency of low concentration Sn doped CdS films offer a good potential as high transparent windows for solar cell applications.

## Figures and Tables

**Table 1:** Details of chemical solutions used in CBD.

System	Volume (mL)			Molar ratio		
	CdSO_4_	Thiourea	SnCl_2_	Cd	S	Sn
1 (undoped)	50	50	0	5	1	0
2	50	50	1	5	1	0.003
3	50	50	3	5	1	0.018
4	50	50	5	5	1	0.030
5	50	50	7	5	1	0.042

**Table 2:** XRD data of Sn:CdS films.

System	*d* spacing of plane (111) (JCPDS) (Å)	*d* spacing of plane (111) (Å)	Crystallite size (nm)
1 (undoped)	3.35	3.35	18.06
2	3.35	3.33	21.68
3	3.36	3.32	27.10
4	3.35	3.31	36.14
5	3.35	3.30	37.09
